# Dendritic-cell depletion and dysfunction in sepsis: a compartment- and evidence-aware synthesis

**DOI:** 10.3389/fimmu.2026.1934483

**Published:** 2026-09-03

**Authors:** Xiao Xu, Maoting Tang, Zhuan Zou, Wanling Zhao, Haiyang Zhang

**Affiliations:** 1 Department of Pediatrics, West China Second University Hospital, Sichuan University, Chengdu, China; 2 Key Laboratory of Birth Defects and Related Diseases of Women and Children, Sichuan University, Ministry of Education, Chengdu, China; 3 Department of Outpatient Children’s Hospital of Chongqing Medical University, National Clinical Research Center for Children and Adolescents’ Health and Diseases, Chongqing, China; 4 Department of Emergency, West China Second University Hospital, Sichuan University, Chengdu, China; 5 NHC Key Laboratory of Chronobiology, Sichuan University, Chengdu, China

**Keywords:** biological compartment, cellular identity, dendritic cells, depletion, immune dysfunction, sepsis, single-cell RNA sequencing

## Abstract

Sepsis-associated immune dysfunction varies across patients, compartments, and sampling windows. Dendritic cells (DCs) offer a useful—but neither exclusive nor primary—lens because they connect innate sensing with antigen presentation, cytokine instruction, and adaptive immunity. Human studies show reduced circulating DC abundance, altered phenotype, checkpoint context, and impaired ex vivo responsiveness, whereas experimental studies demonstrate splenic DC loss, disturbed bone-marrow generation, impaired anigen-specific T-cell priming, and tissue-specific pDC vulnerability or rescue. These observations support four distinct, potentially coexisting contributions: depletion, dysfunction of residual cells, redistribution, and impaired replacement. A low blood count cannot distinguish among them. Across mechanisms, evidence for developmental supply, survival and cell death, trafficking, antigen presentation, cytokine output, regulatory-like programming, checkpoint signaling, and organelle or metabolic stress varies substantially in identity confidence, model, functional validation, and causal support. Accordingly, human blood evidence is not treated as equivalent to murine marrow, spleen, or lung perturbation, and transcriptomic APC/DC-like states are not equated with demonstrated cellular function. The mouse mregDC-like program remains a Class 4 exploratory regulatory-like state with substantial within-study support but no validated human sepsis homolog. Apparent conflicts—including early inflammatory amplification versus persistent immune suppression, and IL-3-associated inflammation versus late pDC-dependent antiviral rescue—are interpreted as context-, time-, and compartment-dependent rather than forced into a universal sequence. DC-informed monitoring should complement established broader approaches such as monocyte HLA-DR and transcriptomic immune subgrouping, using a transparent ten-domain appraisal of identity and evidence. No DC-only endotype, treatment trigger, bedside biomarker, or DC-directed therapy is validated. This synthesis defines testable, timing-aware, and safety-bounded research hypotheses while keeping clinical claims proportional to the evidence.

## 1 Why dendritic-cell evidence matters in sepsis immune dysfunction

Sepsis is clinically defined by life-threatening organ dysfunction caused by a dysregulated host response to infection, but patients meeting that definition do not share a uniform immune state (1). Pathogen class, infection source, source control, sampling day, organ injury, treatment exposure, baseline immune fitness, secondary infection, and recovery course all shape the immune measurements obtained from a cohort. Reviews of sepsis-induced immunosuppression and immunoparalysis caution against reducing this heterogeneity to a uniform progression from hyperinflammation to suppression (2, 3). Human blood single-cell profiling further demonstrates coordinated but nonuniform immune states (4). Disease-stage and gene-expression subgroup studies likewise identify clinically distinct immune patterns rather than a single shared state (5–7).

DCs are useful in this heterogeneous setting because they connect innate sensing with adaptive immune instruction through antigen presentation, costimulation, cytokine production, trafficking, and interactions with T cells. This focus does not imply that DCs are the primary drivers of sepsis pathogenesis, which involves multiple immune, endothelial, coagulation, metabolic, and organ-specific processes. The value of a DC-focused review is therefore organizational rather than causal: relevant findings remain fragmented across depletion, cellular identity, marrow, blood and tissue compartments, sampling time, residual-cell function, developmental supply, and distinct human observational, transcriptomic, and preclinical evidence modalities.

Established human studies document reduced circulating DC abundance and abnormalities in blood DC phenotype or ex vivo responsiveness (8, 9). Phenotypic and functional measurements further show that the remaining blood DC compartment cannot be characterized by count alone (10, 11). Human single-cell studies identify additional APC/DC-associated transcriptional programs in blood, including anergic-like and prognosis-associated states, but primarily provide observational state evidence (4, 12, 13). Mouse models supply complementary access to marrow and multiorgan perturbational mechanisms that are rarely accessible in patients (14, 15). A pediatric human blood/progenitor cohort with *in vitro* differentiation adds supply-side evidence, but it did not directly sample patient marrow (16).

**Figure 1:**
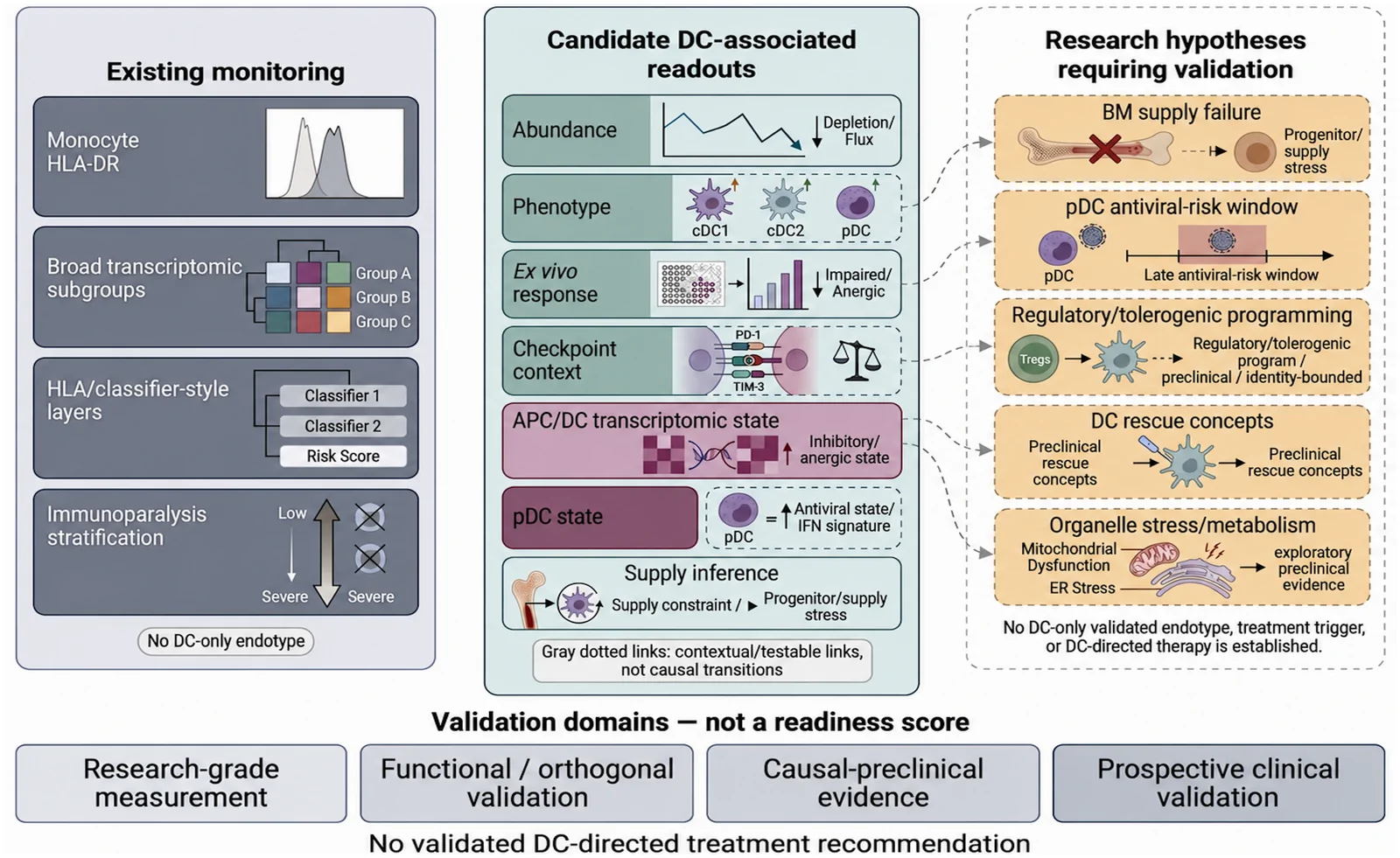
Sepsis-associated DC depletion and evidence domains with distinct support. Depletion is shown separately in human blood and mouse lymphoid/tissue niches and is interpreted through six evidence dimensions: abundance, cell identity, transcriptome, function, compartment, and time/study window. The right-hand domains distinguish residual-cell dysfunction, altered generation/supply, an exploratory preclinical regulatory-like program, pDC observations, and evidence-limited DC–T-cell instruction. These domains may coexist or diverge and should not be read as stages through which the same DCs or patients necessarily pass. Teal solid links denote human blood observations, ochre solid links denote mouse/tissue evidence, and gray dashed links denote cross-study integration; dashed links do not indicate a directly observed transition or causal relationship. DC, dendritic cell; pDC, plasmacytoid dendritic cell.

The evidence spans separable dimensions of established DC dysfunction: abundance and survival; residual phenotype and function; redistribution and localization; progenitor injury and replacement; antigen presentation and costimulation; cytokine and antiviral instruction; regulatory or checkpoint-associated programming; cellular stress; and consequences for DC–T-cell communication. This organization overlaps substantially with immunoparalysis and immune reprogramming, whereas emergency myelopoiesis addresses one supply-side process and tolerogenic or regulatory DC states describe selected functional programs. It does not define a separate category of sepsis biology. These evidence domains and their interpretive boundaries are summarized in **Figure 1**.

After describing how the literature was identified and appraised, the Review defines the cellular-identity and functional terminology used throughout. The core evidence is then organized around depletion, residual dysfunction, redistribution, impaired replacement, single-cell findings, and a time- and compartment-indexed evidence map. Subsequent sections address mechanisms, DC–T-cell circuitry, monitoring, therapeutic hypotheses, and limitations.

## 2 Review approach and evidence appraisal

This narrative/integrative review followed a section-specific search plan organized around sepsis stage and compartment, dendritic-cell (DC) identity, cellular function, mechanism, and translational relevance. The retained search handbook specified PubMed, Web of Science, Google Scholar, and Europe PMC as the principal bibliographic platforms; GEO, SRA, and CellxGene as supplementary sources for single-cell datasets; and ClinicalTrials.gov for translational context. Search concepts combined sepsis or septic shock—including polymicrobial/cecal ligation and puncture models, late sepsis, and chronic critical illness—with DC/APC terms, including cDC1, cDC2, pDC, moDC, DC3, anergic APC, and mregDC/LAMP3 or regulatory-like programs. Additional terms addressed antigen presentation, costimulation, checkpoint signaling, cytokine instruction, DC–T-cell interactions, marrow/progenitor biology, signaling pathways, immune monitoring, secondary infection, and candidate interventions.

Retained project records document that search materials and candidate retrieval records were consolidated on June 24, 2026. Subsequent project batches included iterative metadata normalization, targeted acquisition of readable full text or official article pages, and structured evidence extraction. Candidate records were deduplicated using DOI, PMID, and title anchors; missing or malformed metadata were left unresolved or flagged rather than inferred. Studies were prioritized for substantive synthesis when they directly addressed DC/APC depletion, identity, differentiation, phenotype, function, compartmental distribution, DC–T-cell circuitry, or a non-DC process explicitly affecting those features. Human, mouse, single-cell, mechanistic, translational, and hypothesis-generating evidence were kept distinct. Purely algorithmic predictions, records without DC-specific data, mismatched or unreadable full texts, unverifiable bibliographic identities, and cluster-based functional claims lacking orthogonal support were down-ranked or excluded from load-bearing claims.

Evidence was appraised with a review-specific, non-numerical multidomain scheme. Each claim-bearing study was characterized by cellular identity confidence; species; biological compartment; study design; independent replication; longitudinal support; orthogonal or functional validation; perturbational causal support; cross-species concordance; and clinical relevance/readiness. Cellular identity was classified as: (1) a phenotypically or ontogenetically well-defined DC population; (2) a DC-enriched or historically defined population with limited modern identity resolution; (3) a transcriptomically inferred APC/DC-like state; or (4) a preclinical regulatory/tolerogenic-like or mregDC-like program without an established human homolog. Transcriptomic cluster names were not treated as proof of function, lineage, or temporal transition, and mouse, blood, tissue, marrow, and *in vitro* findings were not merged without explicit qualification.

The synthesis is narrative and integrative rather than an exhaustive systematic review. The retained records do not establish database-specific execution dates and hit counts, a formal language or publication-date restriction, duplicate independent screening, PRISMA compliance, prospective protocol registration, or a formal risk-of-bias assessment; none of these procedures is claimed.

## 3 Defining DC identity, transcriptomic state, and functional evidence

Interpretation begins with the identity of the measured cells. A subset is an operational population defined by markers or clustering; lineage requires developmental or ontogenetic support; a transcriptomic state is an RNA-defined condition at sampling; and a functional state requires evidence that cells execute, or fail to execute, a biological task. These terms are related but not interchangeable. The appraisal scheme therefore places every claim-bearing population into one of four identity classes and displays the supporting evidence domains rather than compressing them into a numerical score.

Class 1 comprises phenotypically or ontogenetically well-defined DC populations. Ontogeny-based nomenclature and human-subset synthesis distinguish cDC1, cDC2, pDC-related, and pre-DC populations (17, 18). The human DC developmental program provides lineage context beyond conventional myeloid-versus-lymphoid labels (19). Comparative transcriptional analysis demonstrates related but non-identical mouse and human DC heterogeneity (20). pDC-like/pre-DC2 lineage work and cDC2 ontogenetic studies further show why partial surface or RNA signatures do not establish lineage identity (21, 22). These ontology studies define interpretive boundaries; they do not retroactively assign precise modern identities to older sepsis cohorts.

Class 2 comprises DC-enriched or historically defined populations with limited modern identity resolution. Older human sepsis studies commonly used lineage-negative, HLA-DR-positive flow gates with CD11c-, CD33-, or CD123-adjacent markers. Those studies remain valid for claims about circulating DC-enriched abundance, broad subset phenotype, and measured ex vivo responses (10, 11). Their results should not be rewritten as exact cDC1, cDC2, DC3, or committed-lineage findings. The same caution applies to broad murine CD11c-positive populations when ontogeny or contamination by other APCs was not resolved.

Class 3 comprises transcriptomically inferred APC/DC-like states. Human blood scRNA-seq can identify broad immune-cell states and anergic-like or prognosis-associated APC/DC programs (4, 12, 13). High-dimensional mass cytometry adds protein/phenotype-level immune architecture but is not transcriptomic evidence (23). Reduced antigen-presentation or costimulatory transcripts support a transcriptional antigen-presentation-state claim; they do not alone demonstrate defective peptide presentation, cytokine secretion, migration, immunological-synapse formation, or antigen-specific T-cell priming.

Class 4 comprises preclinical regulatory/tolerogenic-like or mregDC-like programs without an established human homolog. The label denotes a program defined in a model and does not establish a new lineage, a universal endpoint, or cross-species equivalence. Regulatory or suppressive function may be described only to the depth directly tested. The Yao et al. mouse CLP program is therefore discussed as an mregDC-like regulatory program with multimodal within-study support and a bounded functional interpretation, not as a validated human sepsis subset (15).

Functional labels are reserved for direct or orthogonally supported evidence. Human ex vivo stimulation supports impaired responsiveness when cytokine output or costimulatory phenotype was measured (10). A non-sepsis human blood DC study defines Th1/Th17-instruction capacity, whereas experimental SIRPα–CD47 perturbation clarifies receptor–ligand control of DC–T-cell priming; neither replaces sepsis-specific human functional data (24, 25). In murine sepsis, DC transfer altered T-cell differentiation and survival outcomes (26), DC-derived IL-12 was linked to type-1 instruction (27), and MMP8 perturbation altered late tolerogenic programming (28). A pediatric human cohort instead combined serial blood/progenitor measurements with *in vitro* differentiation and mediator perturbation (16). Species, compartment, cellular definition, and assay therefore remain part of every functional claim.

This hierarchy separates cellular designation from demonstrated function. It also provides the terminology needed to evaluate low DC counts, residual dysfunction, and replacement failure without assuming equivalence among measured populations. **Table 1** summarizes the identity classes and their permitted uses.

**Table 1:** Cellular identity classes, evidence roles, and boundaries for sepsis-associated DC studies.

Evidence role/population	Identity class and definition	Species/compartment	Design and support profile	Supported inference and boundary	Specific refs
Ontology/context: Contemporary DC ontology and nomenclature	Class 1 — phenotypically/ontogenetically well-defined DC	mixed; not sepsis-specific	ontology/review synthesis. Replication: not applicable; longitudinal: not applicable; functional: not applicable; causal: not applicable.	Supported: Defines the criteria used to recognize identity-resolved DC populations. Boundary: Ontology references do not establish a sepsis phenotype or function. Cross-species: ontology compared across species. Clinical: context only.	(17, 18)
Ontology/context: Developmental and transcriptional identity context	Class 1 — phenotypically/ontogenetically well-defined DC	mixed; developmental tissues; not sepsis-specific	developmental/ontogenetic and comparative transcriptomic studies. Replication: independent studies address complementary identity questions; longitudinal: not applicable; functional: identity validation, not sepsis function; causal: study-specific developmental perturbation.	Supported: Supports lineage-aware interpretation beyond CD11c or HLA-DR alone. Boundary: Does not establish abundance, dysfunction, or trajectory in sepsis. Cross-species: cross-species comparison in Brown et al. Clinical: context only.	(19, 20)
Ontology/context: cDC2 and pDC-like lineage refinement context	Class 1 — phenotypically/ontogenetically well-defined DC	mixed; homeostatic tissues; not sepsis-specific	ontogenetic/phenotypic studies. Replication: two independent contextual studies; longitudinal: not applicable; functional: identity/lineage validation; causal: study-specific genetic support.	Supported: Illustrates why modern cDC2 and pDC-like labels require lineage-resolving evidence. Boundary: Cannot be retrofitted onto older sepsis gates. Cross-species: not a sepsis concordance test. Clinical: context only.	(21, 22)
Sepsis-specific evidence: Historically gated circulating MDC/cDC and pDC abundance	Class 2 — DC-enriched or historically defined population	human; blood	observational cohorts using older lineage-negative/HLA-DR and subset gates. Replication: separate cohorts provide cross-study association support; longitudinal: serial sampling in some cohorts; not uniform; functional: none or phenotype only; causal: none.	Supported: Supports reduced or persistently altered circulating DC-enriched abundance. Boundary: Does not resolve modern cDC1/cDC2/DC3 identity or distinguish death, redistribution, and impaired production. Cross-species: not assessed. Clinical: human observational discovery/research monitoring.	(8, 9, 11)
Sepsis-specific evidence: Historically gated circulating MDC/PDC phenotype and ex vivo response	Class 2 — DC-enriched or historically defined population	human; blood	prospective observational cohort with flow cytometry and ex vivo stimulation. Replication: no matched independent functional replication established here; longitudinal: baseline and day-28 sampling; functional: direct ex vivo cytokine-response assays; causal: none.	Supported: Supports coexistence of low blood abundance, altered HLA-DR phenotype, and impaired measured responsiveness in a selected cohort. Boundary: Does not prove tissue DC dysfunction or antigen-specific human DC–T-cell synapse failure. Cross-species: not assessed. Clinical: human ex vivo research evidence.	(10)
Sepsis-specific evidence: Human anergic-like APC/DC transcriptomic state	Class 3 — transcriptomically inferred APC/DC-like state	human; blood	cross-sectional scRNA-seq/reanalysis with protein-marker confirmation. Replication: no matched independent functional replication assigned; longitudinal: no repeated-participant trajectory established; functional: marker/protein confirmation; no direct antigen-presentation assay; causal: none.	Supported: Supports an RNA-defined antigen-presentation-low/anergic-like APC/DC state. Boundary: Cluster naming and RNA programs do not prove lineage, anergy, or T-cell priming failure. Cross-species: not assessed. Clinical: human observational discovery.	(12)
Sepsis-specific evidence: Broader human blood APC/DC transcriptomic states	Class 3 — transcriptomically inferred APC/DC-like state	human; blood	single-cell observational cohort/reanalysis. Replication: different cohorts provide contextual convergence, not matched replication of one state; longitudinal: study-dependent; no universal repeated-patient sequence; functional: omics; functional validation variable or absent; causal: none.	Supported: Supports heterogeneity of APC/DC-associated transcriptional programs across clinical cohorts. Boundary: Does not establish one DC taxonomy, function, or continuous temporal trajectory. Cross-species: contextual human concordance only. Clinical: human observational discovery.	(4, 13)
Sepsis-specific evidence: Mouse mregDC-like regulatory program	Class 4 — preclinical regulatory/mregDC-like program without established human homolog	mouse; multi-compartment; key functional work in spleen	CLP serial cross-sectional scRNA/omics plus protein/spatial, sorted-cell co-culture, and inhibitor experiments. Replication: external-dataset resemblance; no matched independent replication; longitudinal: independent animals at 8, 24, and 72 h; pseudotime inferential; functional: flow/spatial confirmation and sorted-cell CD4 T-cell co-culture; causal: pathway-inhibitor and source-population support; no selective *in vivo* mregDC depletion.	Supported: Supports an exploratory mouse regulatory-like program with mixed T-cell-instructive effects *in vitro*. Boundary: Not a validated human subset, same-cell transition, universal endpoint, or clinically actionable target. Cross-species: transcriptomic resemblance only; no human sepsis homolog. Clinical: preclinical discovery.	(15)
Sepsis-specific evidence: Circulating pDC abundance/time associations	Class 2 — DC-enriched or historically defined population	human; blood	observational pDC flow cohorts. Replication: separate cohorts; matched replication not established; longitudinal: serial course in Weber et al.; outcome association in separate cohort; functional: no direct antiviral functional assay in these rows; causal: none.	Supported: Supports time- and outcome-associated variation in circulating pDC measures. Boundary: Blood percentages do not establish lung recruitment, antiviral function, or a treatment trigger. Cross-species: not assessed. Clinical: human observational discovery/research monitoring.	(29, 30)
Sepsis-specific evidence: Post-sepsis lung pDC recruitment and antiviral priming	Class 2 — DC-enriched or historically defined population	mouse; lung	CLP plus secondary viral-pneumonia perturbation. Replication: no matched independent replication assigned; longitudinal: defined post-sepsis challenge window; not same-cell tracking; functional: pDC recruitment and T-cell priming measured; causal: IL-3/genetic-intervention support within model.	Supported: Supports a compartment-specific pDC-dependent antiviral rescue mechanism in mice. Boundary: Not evidence for a human pDC homologous trajectory or clinical IL-3 use. Cross-species: human blood pDC evidence is associative and not functionally matched. Clinical: preclinical discovery.	(31)

## 4 DC depletion, residual dysfunction, redistribution, and impaired replacement

Reduced DC abundance is the most established entry point to sepsis-associated DC dysfunction. In human septic shock, lower circulating DC numbers were associated with fatal outcome, and persistent reduction was linked to ICU-acquired infection (8, 9). These studies establish a clinically visible blood signal. They do not identify whether the signal reflects cell death, tissue trafficking or retention, reduced marrow output, altered marker expression, or a combination of processes.

True depletion or death is most directly supported in experimental lymphoid tissue. Sepsis-induced apoptosis and marked loss of splenic interdigitating and follicular DCs showed that DCs can be removed from the architecture in which antigen capture and T-cell instruction occur (32). TLR2- and TLR4-associated effects on splenic DC depletion and maturation or survival signaling provide perturbational support for specific pathways in murine models (33). These findings establish a tissue depletion mechanism in mice, not the cause of every low human blood count.

Residual-cell dysfunction is a separate contributor. Human studies reported altered phenotype and impaired ex vivo responsiveness in the blood DC-enriched populations that remained measurable during sepsis (10, 11). Thus, numerical preservation would not guarantee intact antigen presentation, costimulation, or cytokine instruction, and numerical loss does not specify the state of surviving cells. Transcriptomic evidence adds candidate residual APC/DC-like programs, but direct function must be assigned from the assay rather than inferred from an RNA label (12).

Redistribution is a third possible explanation for compartment-specific abundance changes. Blood is a sampling compartment, not a census of marrow, spleen, lung, lymph nodes, or infected tissues. Movement into or retention within tissues, altered egress, vascular or inflammatory trafficking, and changes in compartmental residence can therefore contribute to discordant blood and tissue measurements. The current evidence base does not quantify redistribution sufficiently to treat it as the universal explanation for circulating depletion; it should be retained as a testable alternative whenever blood counts are interpreted.

Impaired generation or replacement provides a fourth, supply-side mechanism. In murine polymicrobial sepsis, altered bone-marrow DC differentiation was associated with sustained immunosuppression (14). Mouse systemic bacterial-infection models also show emergency monopoiesis at the expense of DC differentiation, a supply-side pattern reviewed across related infection settings (34, 35). TLR2-induced CD8 T-cell deactivation provides perturbational evidence that adaptive immune changes can feed back on murine marrow DC differentiation (36). In a pediatric human cohort, serial blood and progenitor measurements plus *in vitro* differentiation implicated inflammation-associated progenitor injury, but patient marrow was not directly sampled (16). Thus, impaired replacement is mechanistically supported in mice and indirectly supported in human blood/progenitor assays; a corresponding *in vivo* human marrow mechanism has not been established.

The functional importance of loss and dysfunction is supported most directly by mouse experiments. Polymicrobial sepsis reduced DC number and function and impaired primary CD8 T-cell responses *in vivo* (37). This result links an altered DC compartment to an antigen-specific adaptive output, but it does not resolve how much of the effect arose from cell loss versus dysfunction of remaining cells. It also cannot be substituted for direct human antigen-specific functional evidence.

The four contributors can coexist and may dominate in different compartments or sampling windows. A low blood count can accompany true tissue loss, redistribution, weak developmental replacement, or residual dysfunction; no single measurement distinguishes them. The next analytical step is therefore to examine disease-associated APC/DC transcriptomic states while preserving the identity and functional limits defined above.

## 5 Single-cell and transcriptomic evidence for disease-associated APC/DC states

Single-cell studies extend the evidence beyond compartment-average counts by resolving cellular composition and RNA programs at sampling. Their principal contribution is not proof of a unified DC sequence, but identification of residual or emergent APC/DC-like states that can be compared with protein, functional, perturbational, and clinical evidence. Most human sepsis single-cell findings fall within identity Class 3 unless a population is independently resolved by phenotypic or ontogenetic evidence.

Reyes et al. defined coordinated immune-state changes in human bacterial sepsis blood, providing broad context for APC/DC remodeling rather than a DC-specific functional test (4). More DC-focused analysis identified anergic-like antigen-presenting-cell subtypes with suppressed antigen-presentation and activation programs (12). Prognosis-stratified data further indicate that circulating immune-state composition and transcription differ across clinical outcomes (13). These studies support observational human blood transcriptomic-state claims; they do not establish tissue-resident DC dysfunction or direct antigen-specific failure.

“Anergic-like” is used here as a transcriptomic qualifier rather than a demonstrated cellular function. Lower expression of antigen-presentation, costimulatory, or inflammatory-response programs is compatible with reduced immune instruction, but RNA abundance is not equivalent to antigen processing, peptide loading, protein expression, cytokine secretion, migration, or T-cell priming. The functional claim becomes stronger only when the same population is linked to orthogonal protein measurements, stimulation assays, sorted-cell experiments, or perturbation.

The mouse CLP study by Yao et al. identified an mregDC-like regulatory program using scRNA-seq with flow-cytometric and spatial confirmation, sorted-cell CD4 T-cell co-culture, and pathway-inhibitor experiments (15). These assays provide multimodal within-study support for T-cell-instructive and regulatory-like activity *in vitro*, although the functional pattern was not purely suppressive. Samples obtained from separate animals at 8, 24, and 72 hours provide serial cross-sectional evidence rather than same-cell or repeated-animal tracking. No matched independent replication, selective *in vivo* depletion of the mregDC-like population, or validated human sepsis homolog was verified. The appropriate classification is therefore identity Class 4: a preclinical, exploratory, hypothesis-generating regulatory-like program for human sepsis.

The mregDC-like mouse program should not be equated with the human anergic-like APC/DC state. The two observations differ in species, compartment, model, identity assignment, and functional validation. Reanalysis showing transcriptomic resemblance in another inflammatory context can motivate comparison, but the absence of a matched human sepsis population prevents a claim of cross-species conservation. Any temporal ordering based on pseudotime or separate sampled animals remains an origin or differentiation hypothesis rather than a directly observed continuous transition.

Late and persistent sepsis datasets add time-window information without proving continuity. Non-myeloid single-cell analysis in late sepsis and persistent post-sepsis immune signatures show that blood immune abnormalities can remain after the acute insult (38, 39). Pediatric single-cell data provide an additional clinical context but should not be merged uncritically with adult cohorts (40). These studies support age- and window-specific observations, not a shared endpoint for all patients.

pDC findings further demonstrate why abundance, transcription, and function must remain separate. Human studies support time-associated changes in circulating pDC abundance and survival-related associations (29, 30). In a distinct preclinical setting, IL-3 enhanced lung pDC recruitment and T-cell priming during post-sepsis viral pneumonia (31). The human observations do not prove impaired antiviral priming, and the mouse lung rescue does not establish an IL-3-responsive defect or treatment indication in patients.

Together, single-cell data refine established DC dysfunction by revealing heterogeneity within the residual APC/DC compartment. The evidence supports multiple disease-associated programs rather than a single conserved sepsis DC state. Their biological interpretation requires identity-resolved protein validation, direct functional assays, clinically relevant outcomes, and sampling designs that distinguish within-person change from differences among cohorts.

## 6 Time- and compartment-indexed evidence across marrow, blood, lymphoid tissue, and lung

Time and compartment determine what each DC observation can establish. A blood count during septic shock, a splenic apoptosis measurement after cecal ligation and puncture, a marrow differentiation experiment, a tissue single-cell profile, and a lung pDC perturbation do not measure the same biological event. We therefore use a working evidence map that records the sampled window and compartment and labels relationships as longitudinally observed, serial cross-sectional, perturbationally supported, or inferred across studies.

Human blood provides the most accessible clinical window. Reduced circulating DC abundance and outcome-associated persistent depletion were directly observed in patient cohorts (8, 9). Separate flow and ex vivo studies documented altered phenotype, subset distribution, and residual responsiveness (10, 11). Repeated sampling can establish within-person or cohort-level change when the design supports it, but blood measurements cannot by themselves identify splenic loss, tissue recruitment, or marrow output. Low blood abundance should therefore be interpreted as a compartment-specific signal with several possible biological sources.

Bone-marrow evidence addresses developmental supply. Murine studies directly support altered marrow DC differentiation after polymicrobial sepsis, emergency monopoiesis at the expense of DC differentiation, and T-cell feedback onto DC generation (14, 34, 36). A pediatric human study adds serial blood/progenitor measurements and *in vitro* differentiation evidence but does not directly establish an *in vivo* marrow trajectory (16). These findings support a plausible route to prolonged peripheral abnormalities. The connection from experimental marrow mechanisms to a patient’s serial blood DC recovery remains cross-study inferred until paired human supply markers or marrow/progenitor measurements are available.

Spleen and lymphoid-tissue studies provide perturbational and tissue-level evidence for depletion, survival signaling, and consequences for adaptive priming. Murine splenic DC apoptosis or depletion and TLR2/TLR4 dependence establish mechanisms within lymphoid architecture (32, 33). Impaired primary CD8 T-cell responses after polymicrobial sepsis provide a functional link between the altered DC compartment and adaptive immunity (37). Neither result establishes that circulating human DC measurements report the same tissue process.

Lung and pDC evidence defines a separate late antiviral hypothesis. In a post-sepsis viral-pneumonia model, IL-3-dependent enhancement of lung pDC recruitment and T-cell priming is perturbationally supported (31). Human blood pDC changes may justify parallel monitoring, but a relation between circulating pDCs, lung recruitment, and antiviral function remains unproven in patients. The lung module should therefore be displayed beside, not downstream of, blood and marrow observations.

Single-cell studies contribute differently to the map. Human blood datasets provide clinical transcriptomic states at defined sampling windows (4, 12, 13). Persistent and pediatric datasets add distinct late or age-specific contexts (39, 40). The Yao et al. mouse atlas samples several compartments and time points, but its 8-, 24-, and 72-hour observations are serial cross-sectional because different animals were used (15). Pseudotime and alignment across these datasets remain inferential. .

No study set reviewed here demonstrates a continuous sequence from early depletion through marrow failure to anergic-like or regulatory-like programming and late pDC vulnerability in the same cells or patients. Accordingly, arrows connecting these observations denote the type of support rather than biological inevitability. Longitudinally observed links require repeated measurements in the relevant subjects; serial cross-sectional links compare independently sampled time points; perturbational links require experimental manipulation; and cross-study links remain hypotheses assembled from non-identical populations, compartments, or models.

**Figure 2:**
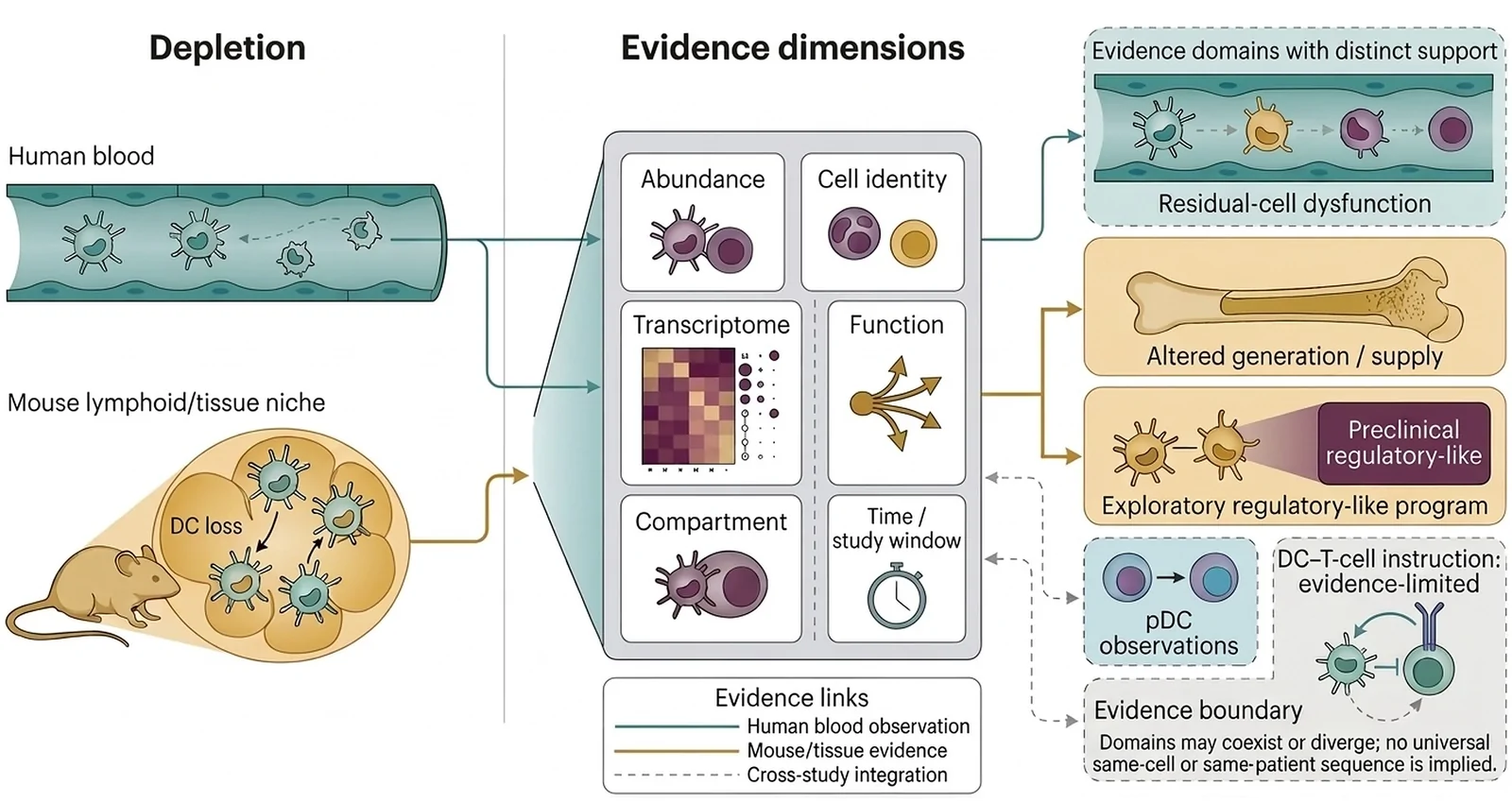
Time- and compartment-indexed evidence map for sepsis-associated DC findings. The map arranges observations across bone marrow, blood, lymphoid/tissue, and lung compartments and across acute, evolving, late, and persistent study windows. Teal identifies human blood monitoring, whereas ochre identifies mouse/tissue mechanistic evidence. A teal line with open circles denotes a longitudinally observed relationship, an ochre arrow denotes a perturbationally supported relationship, and a gray dashed arrow denotes a relationship inferred across studies. The map integrates evidence on bone-marrow supply failure and progenitor bottlenecks, emergency myelopoiesis, exploratory regulatory-like states, blood DC abundance/phenotype and ex vivo response, longitudinal pDC observations, APC/DC transcriptomic states, splenic depletion, tissue remodeling and trafficking, and a testable late pDC antiviral module. The columns represent sampled evidence windows rather than standardized clinical stages, and the arrangement does not imply a universal same-cell or same-patient trajectory. APC, antigen-presenting cell; BM, bone marrow; DC, dendritic cell; pDC, plasmacytoid dendritic cell.

This evidence map translates into a concrete study-design requirement. Future cohorts should combine serial identity-resolved blood DC measurements with phenotype and ex vivo function, clinically feasible indicators of developmental supply, tissue or tissue-adjacent sampling when justified, and outcomes such as immune recovery, secondary infection, viral reactivation, or survival. Accordingly, **Figure 2** displays compartments, sampling windows, and relation labels without presenting a validated patient trajectory. With these boundaries established, the next section examines mechanisms that alter DC generation, survival, and immune function.

## 7 Mechanisms affecting DC generation, survival, trafficking, and function

Systemic infection, tissue injury, microbial products, inflammatory mediators, and altered hematopoietic signals can affect DC biology at several distinct points: developmental supply, survival, localization, antigen presentation, cytokine instruction, regulatory programming, checkpoint context, and cellular stress responses. Each perturbation is therefore described according to the outcome actually measured, such as cell number, phenotype, cytokine output, antigen-specific T-cell priming, tissue recruitment, or survival in an experimental model. Interpretation also considers cellular identity, species, compartment, design, longitudinal support, functional validation, perturbational support, cross-species concordance, and clinical relevance. This structure prevents a pathway name, RNA signature, or outcome association from being treated as a complete mechanism.

Developmental supply is supported most directly by mouse bone-marrow studies. After polymicrobial sepsis, altered progenitor differentiation and impaired reconstitution reduced the generation of immunocompetent DCs (14). Systemic bacterial inflammation also favored emergency monopoiesis at the expense of DC differentiation (34). TLR2-associated CD8 T-cell deactivation further changed marrow DC differentiation, showing that an upstream adaptive perturbation can feed back onto the DC supply line (36). In a pediatric human cohort, inflammation-associated progenitor injury was evaluated through serial blood/progenitor measurements and *in vitro* differentiation rather than direct patient-marrow sampling (16). The measured consequence across these studies is altered output or function of newly generated DCs, not simply disappearance of mature blood cells. The mouse perturbations justify impaired replacement as one contributor to persistent DC abnormalities, whereas the human study supplies indirect progenitor-level concordance; neither establishes a clinical marrow treatment trigger.

Survival and cell death provide a distinct route to quantitative loss. In murine sepsis, apoptosis and profound depletion of splenic interdigitating and follicular DCs directly reduced DC abundance within lymphoid architecture, and TLR2/TLR4 perturbation implicated innate-receptor signaling in this tissue loss (32, 33). Sestrin2 studies subsequently linked stress protection to reduced endoplasmic-reticulum-stress apoptosis or DC pyroptosis and to improved outcomes in experimental sepsis (41, 42). These studies support causal or mechanistic relationships within their mouse or experimental systems, but they do not show that apoptosis accounts for every low circulating DC count in patients. Cell death must therefore remain separate from redistribution, marker change, and impaired replacement.

Trafficking and compartmental residence can change where DCs and their immune effects are observed without proving whole-body depletion. Blood measurements cannot determine whether cells have entered, remained within, or failed to exit infected tissues or lymphoid sites. Preclinical studies show that DC-associated signaling can shape tissue inflammatory-cell recruitment and systemic inflammation: DC-specific TLR4–IL-10 signaling affected neutrophil CXCR2-dependent recruitment, bacterial clearance, and survival, whereas DC PAR1–S1P3 signaling coupled coagulation to inflammatory amplification (43, 44). These perturbations establish functional consequences in experimental tissue or systemic circuits, but they neither quantify DC redistribution in human sepsis nor justify using a blood DC decrease as a direct tissue-trafficking marker.

Antigen presentation and checkpoint context link an altered DC compartment to adaptive immune instruction. Human blood studies document phenotype changes and impaired ex vivo responsiveness in DC-enriched populations, while increased PD-1/PD-L1 expression on circulating antigen-presenting cells was associated with early organ dysfunction and mortality (10, 45). These clinically relevant observational or ex vivo findings do not directly measure antigen-specific DC–T-cell interactions in lymphoid tissue. In mice, polymicrobial sepsis impaired primary CD8 T-cell responses through reduced DC number and function, and transfer of bone-marrow-derived DCs altered inhibitory or regulatory T-cell differentiation and improved survival (26, 37). Together, these findings support the inference that DC antigen-presentation state can alter adaptive output. However, neither APC checkpoint expression nor murine DC rescue validates a DC-specific checkpoint treatment in patients.

Cytokine instruction can be impaired even when residual DCs remain detectable. Severe innate stimulation produced epigenetic suppression of DC-derived IL-12, with a measured loss of Th1-instructive capacity in experimental systems (27). pDC-related cytokine biology illustrates why context and timing cannot be collapsed. IL-3 amplified acute inflammation in sepsis models, whereas it enhanced lung pDC recruitment and antiviral T-cell priming in a post-sepsis viral-pneumonia model (31, 46). These findings concern different perturbations and disease windows rather than opposite measurements of one universal effect. They support a timing- and compartment-dependent IL-3/pDC hypothesis, not an unqualified immune-restoration strategy.

Regulatory or tolerogenic-like programming may redirect DC instruction rather than merely reduce it. In mouse CLP, the mregDC-like program showed transcriptomic, protein/spatial, sorted-cell CD4 T-cell co-culture, and pathway-inhibitor support, but its functional pattern was mixed rather than uniformly suppressive (15). MMP8 perturbation linked late DC tolerance to NF-κB p65/β-catenin signaling, and experimentally generated regulatory DCs restrained acute lethal systemic inflammation in another preclinical setting (28, 47). These studies justify a preclinical regulatory-like program with measured T-cell or inflammatory consequences. They do not establish a conserved human mregDC subset, a universal late endpoint, or a population-specific clinical target.

Organelle and metabolic pathways are emerging modifiers of DC fate and responsiveness, not coequal established mechanisms in human sepsis. One preclinical study linked mtDNA–STING signaling to DC immunoparalysis (48), whereas another linked miR-146a-5p/ATG7 perturbation to DC activation and glycolytic remodeling (49). PINK1-mediated mitochondrial quality control was separately associated with protection from DC dysfunction (50). Sestrin2 studies addressed endoplasmic-reticulum-stress apoptosis and DC pyroptosis in distinct experimental settings (41, 42). More recent single-pathway studies nominate RETREG1/FAM134B-mediated ERGICphagy and NUFIP1-mediated ribophagy as modifiers of DC cell death or immune function (51, 52). Collectively, these studies support the limited inference that cellular quality-control systems can modify DC survival and function. Replication, human DC validation, pathway specificity, and clinically feasible measurement remain insufficient for biomarker or target claims. **Table 2** summarizes the biological programs and their evidence boundaries; the next section addresses the functional DC–T-cell circuits in which several of these effects converge.

**Table 2:** Mechanisms affecting DC generation, survival, trafficking, and immune function.

Biological mechanism/DC process	Identity class	Species/compartment	Design and support profile	Measured consequence, inference, and boundary	Specific refs
Lymphoid-tissue depletion and apoptosis	Class 2 — DC-enriched or historically defined population	mouse; spleen	CLP/tissue histology and mechanistic experiments. Replication: classic evidence plus a separate receptor-perturbation study; longitudinal: time-point specific; no continuous tracking; functional: tissue abundance/apoptosis assays; causal: TLR2/TLR4 perturbation in the model.	Supported: Supports splenic DC loss/apoptosis as one cause of reduced DC abundance in mice. Boundary: Does not explain every low circulating human DC count or all tissues. Cross-species: human blood loss is not a matched compartment. Clinical: preclinical discovery.	(32, 33)
ER-stress protection and apoptosis threshold	Class 2 — DC-enriched or historically defined population	mouse; cultured BMDCs/experimental sepsis	pathway-focused experimental study. Replication: no matched independent replication assigned; longitudinal: none; functional: cell-death and stress assays; causal: Sestrin2 manipulation.	Supported: Supports Sestrin2-dependent protection from ER-stress-associated DC apoptosis in the tested model. Boundary: Single-program preclinical evidence; not a clinical target claim. Cross-species: no human DC concordance established. Clinical: preclinical discovery.	(41)
Pyroptosis control and experimental outcome	Class 2 — DC-enriched or historically defined population	mouse; DC-enriched experimental system/systemic sepsis	genetic/pathway perturbation. Replication: no matched independent replication assigned; longitudinal: none; functional: DC pyroptosis and model-outcome assays; causal: Sestrin2 perturbation.	Supported: Supports a Sestrin2-linked DC pyroptosis mechanism with experimental outcome effects. Boundary: Does not establish human pathway readiness or DC-specific clinical causality. Cross-species: no human DC concordance established. Clinical: preclinical discovery.	(42)
Post-sepsis marrow differentiation impairment	Class 2 — DC-enriched or historically defined population	mouse; bone marrow	polymicrobial-sepsis marrow differentiation study. Replication: no matched independent replication assigned; longitudinal: post-sepsis sampling; repeated-animal trajectory not established; functional: newly generated DC phenotype/function assessed; causal: model-level perturbational support.	Supported: Supports impaired DC generation as distinct from mature-cell loss. Boundary: Not proof of a human marrow mechanism or replacement therapy trigger. Cross-species: no matched human marrow confirmation. Clinical: preclinical discovery.	(14)
Human progenitor targeting and impaired DC generation	Class 1 — phenotypically/ontogenetically well-defined DC	human; blood and HSPC culture	pediatric cohort plus *in vitro* HSPC differentiation under inflammatory mediators. Replication: no matched external replication assigned; longitudinal: days 1–7 patient sampling; not lineage tracking; functional: flow phenotype, apoptosis, differentiation, and cytokine assays; causal: mediator exposure *in vitro*.	Supported: Supports inflammatory impairment of DC survival and HSPC-derived DC generation in a defined pediatric cohort and culture system. Boundary: Peripheral progenitor measurements and culture do not establish whole-marrow causality or clinical intervention readiness. Cross-species: human evidence; tissue marrow inference remains indirect. Clinical: human mechanistic/ex vivo research evidence.	(16)
Emergency monopoiesis at the expense of DC differentiation	Class 2 — DC-enriched or historically defined population	mouse; bone marrow	infection/inflammation hematopoiesis experiments. Replication: support across distinct preclinical studies; not a matched replication set; longitudinal: time-point specific; functional: lineage-output assays; causal: experimental inflammatory/pathogen perturbation.	Supported: Supports competition between inflammatory myeloid output and DC differentiation as a supply-side mechanism. Boundary: Does not establish the same lineage allocation in human sepsis. Cross-species: human concordance not established. Clinical: preclinical/contextual.	(35, 34)
CD8 T-cell feedback onto marrow DC differentiation	Class 2 — DC-enriched or historically defined population	mouse; bone marrow	polymicrobial-sepsis mechanistic and cell-interaction experiments. Replication: no matched independent replication assigned; longitudinal: defined post-sepsis windows; no same-cell tracking; functional: DC differentiation and T-cell-state assays; causal: TLR2/CD8 perturbational support.	Supported: Supports T-cell-to-DC feedback that changes future DC supply. Boundary: Must remain distinct from DC-to-T-cell priming and from human marrow claims. Cross-species: no human marrow concordance established. Clinical: preclinical discovery.	(36)
Antigen-presentation phenotype and residual responsiveness	Class 2 — DC-enriched or historically defined population	human; blood	prospective flow cohort with ex vivo stimulation. Replication: no matched functional replication established; longitudinal: baseline/day-28 sampling; functional: direct ex vivo cytokine-response assays; antigen-specific synapse not measured; causal: none.	Supported: Supports impaired residual-cell phenotype and measured responsiveness alongside depletion. Boundary: Does not directly demonstrate human antigen-specific DC–T-cell synapse failure. Cross-species: not assessed. Clinical: human ex vivo research evidence.	(10)
DC-dependent primary CD8 T-cell priming	Class 2 — DC-enriched or historically defined population	mouse; spleen/systemic	CLP with antigen targeting and Flt3L intervention. Replication: no matched independent replication assigned; longitudinal: post-sepsis assessment; no same-cell trajectory; functional: *in vivo* antigen-specific CD8 response and DC cytokine function; causal: DC targeting/Flt3L intervention.	Supported: Supports a causal contribution of DC loss/dysfunction to impaired primary CD8 responses in mice. Boundary: Does not prove feasibility or efficacy of DC-directed rescue in patients. Cross-species: no direct human synapse concordance. Clinical: preclinical discovery.	(37)
DC-derived IL-12 restraint after severe innate activation	Class 2 — DC-enriched or historically defined population	mouse; DC culture/systemic inflammatory models	epigenetic and glucocorticoid/pathway experiments. Replication: related studies address distinct regulatory contexts; longitudinal: time/context dependent; functional: IL-12 and T-cell-instruction assays; causal: pharmacologic/genetic or endocrine perturbation within models.	Supported: Supports context-dependent suppression or restraint of DC-derived IL-12 and type-1 instruction. Boundary: More IL-12 is not automatically beneficial; human treatment relevance is unproven. Cross-species: human sepsis conservation not established. Clinical: preclinical discovery.	(27, 53)
Endotoxin tolerance and tissue cytokine-profile change	Class 2 — DC-enriched or historically defined population	mouse; systemic/lung tissue	experimental tolerance and post-sepsis lung studies. Replication: two distinct models; not matched replication; longitudinal: stage-specific; functional: cytokine and tissue immune-response assays; causal: model perturbation/secondary challenge.	Supported: Supports context-specific loss or redirection of cytokine instruction. Boundary: The studies should not be merged into one universal suppressive sequence. Cross-species: human concordance not established. Clinical: preclinical discovery.	(54, 55)
Mouse mregDC-like regulatory program	Class 4 — preclinical regulatory/mregDC-like program without established human homolog	mouse; multi-compartment; spleen for key function	serial cross-sectional scRNA plus multimodal/function/inhibitor experiments. Replication: no matched independent replication; longitudinal: 8, 24, 72 h in different animals; functional: protein/spatial confirmation and sorted-cell CD4 co-culture; causal: pathway-inhibitor support; no selective *in vivo* depletion.	Supported: Supports an exploratory regulatory-like program with mixed *in vitro* T-cell effects. Boundary: Not a validated lineage, continuous transition, or clinical target. Cross-species: transcriptomic resemblance only; no human sepsis homolog. Clinical: preclinical discovery.	(15)
MMP8-associated tolerogenic DC programming	Class 2 — DC-enriched or historically defined population	mouse; spleen/BMDC; late sepsis	late-CLP experiments with knockout/knockdown, adoptive transfer, and pathway inhibition. Replication: no matched independent replication assigned; longitudinal: day-3/second-hit context; not trajectory tracking; functional: DC phenotype/function, T-cell output, organ injury, and survival; causal: MMP8 perturbation; NF-κB p65/β-catenin-associated pathway support.	Supported: Supports MMP8-linked tolerogenic programming and measured T-cell/output effects in late murine sepsis. Boundary: Does not establish a human target or equivalence to the mregDC-like program. Cross-species: human DC tolerance unvalidated. Clinical: preclinical discovery.	(28)
Experimentally generated regulatory DC control of systemic inflammation	Class 2 — DC-enriched or historically defined population	mouse; systemic/ex vivo-generated regulatory DCs	preclinical cell-transfer/functional study. Replication: no matched independent replication assigned; longitudinal: acute model; functional: *in vivo* inflammatory outcome; causal: cell-transfer perturbation.	Supported: Supports the possibility that regulatory DC programs can modify systemic inflammation in a model. Boundary: Non-sepsis/experimental regulatory DC context; not evidence for a natural human sepsis subset. Cross-species: human sepsis concordance not established. Clinical: preclinical proof-of-principle.	(47)
mtDNA–STING-associated DC immunoparalysis	Class 2 — DC-enriched or historically defined population	mouse/*in vitro*; DC experimental system	single pathway-focused perturbational study. Replication: no matched independent replication assigned; longitudinal: none; functional: DC immune-function assays; causal: STING-pathway manipulation.	Supported: Supports a candidate mtDNA–STING modifier of DC dysfunction in the tested system. Boundary: Single-study pathway; no clinical biomarker or target implication. Cross-species: human DC concordance not established. Clinical: preclinical exploratory.	(48)
miR-146a-5p/ATG7 regulation of DC activation and glycolysis	Class 2 — DC-enriched or historically defined population	human serum; donor-derived DC culture; mouse CLP/lung	serum comparison, LPS-stimulated DC culture, T-cell co-culture, and small CLP study. Replication: no matched independent replication; longitudinal: active-versus-cured groups, not within-patient tracking; functional: DC markers, glycolytic, T-cell, cytokine, and lung assays; causal: miR-146a-5p inhibition and ATG7 overexpression.	Supported: Links miR-146a-5p/ATG7 perturbation to DC activation-associated markers, glycolytic readouts, T-cell responses, and experimental sepsis/lung outcomes. Boundary: DC identity is incompletely resolved; the animal experiment was small; independent replication and human causal validation are lacking. Cross-species: human-associated and mouse findings are from one study. Clinical: exploratory association plus preclinical mechanism.	(49)
PINK1-mediated mitochondrial quality control	Class 2 — DC-enriched or historically defined population	mouse/*in vitro*; DC experimental system	single pathway-focused perturbational study. Replication: no matched independent replication assigned; longitudinal: none; functional: mitochondrial quality/function assays; causal: PINK1 manipulation.	Supported: Supports a candidate mitochondrial-quality-control modifier of DC dysfunction. Boundary: Single-study preclinical evidence; no validated human assay or target. Cross-species: human DC concordance not established. Clinical: preclinical exploratory.	(50)
RETREG1/FAM134B-mediated ERGICphagy	Class 2 — DC-enriched or historically defined population	mouse/*in vitro*; DC experimental system	single pathway-focused perturbational study. Replication: no matched independent replication assigned; longitudinal: none; functional: organelle-stress and immune-function assays; causal: pathway manipulation.	Supported: Supports a candidate ERGICphagy modifier of DC immune function. Boundary: Single-study preclinical evidence; independent replication and human validation lacking. Cross-species: human DC concordance not established. Clinical: preclinical exploratory.	(51)
NUFIP1-mediated ribophagy	Class 2 — DC-enriched or historically defined population	mouse/*in vitro*; DC experimental system	single pathway-focused perturbational study. Replication: no matched independent replication assigned; longitudinal: none; functional: ribophagy and immune-function assays; causal: pathway manipulation.	Supported: Supports a candidate ribophagy modifier of DC immune function. Boundary: Single-study preclinical evidence; independent replication and human validation lacking. Cross-species: human DC concordance not established. Clinical: preclinical exploratory.	(52)
DC-linked inflammatory-cell trafficking	Class 2 — DC-enriched or historically defined population	mouse; peritoneal/tissue	DC-associated signaling perturbation. Replication: no matched independent replication assigned; longitudinal: acute model; functional: PMN recruitment, bacterial clearance, and survival; causal: DC-specific TLR4/IL-10 pathway perturbation.	Supported: Supports a DC-linked mechanism affecting tissue neutrophil trafficking and host defense. Boundary: Not a blood DC monitoring claim or general trafficking rule. Cross-species: human blood concordance not established. Clinical: preclinical discovery.	(43)
PAR1–S1P3 inflammation–coagulation coupling context	Class 2 — DC-enriched or historically defined population	mouse; tissue/systemic	DC-associated receptor-pathway perturbation. Replication: no matched independent replication assigned; longitudinal: acute model; functional: inflammatory/coagulation outcome assays; causal: PAR1/S1P3 perturbation.	Supported: Provides a preclinical example of DC-linked inflammation–coagulation coupling. Boundary: Contextual mechanism; not evidence for a validated sepsis DC monitoring marker. Cross-species: human concordance not established. Clinical: preclinical/contextual.	(44)
Noncanonical DC differentiation and survival context	Class 2 — DC-enriched or historically defined population	mouse; developmental/preclinical tissues	developmental/context studies. Replication: independent studies address different developmental controls; longitudinal: not sepsis longitudinal evidence; functional: lineage output/survival assays; causal: study-specific developmental perturbation.	Supported: Shows that DC supply and tissue homeostasis depend on more than mature-cell survival. Boundary: Contextual unless directly validated in sepsis and the relevant compartment. Cross-species: not tested as human sepsis concordance. Clinical: preclinical context.	(56, 57)
Wnt5a restriction of Flt3+ progenitor expansion	Class 2 — DC-enriched or historically defined population	mouse; bone marrow/developmental	developmental pathway perturbation. Replication: no matched independent replication assigned; longitudinal: not sepsis longitudinal evidence; functional: progenitor expansion/differentiation assays; causal: Wnt5a pathway perturbation.	Supported: Provides a specific developmental constraint relevant to supply-side hypotheses. Boundary: Not direct evidence that Wnt5a drives impaired DC replacement in sepsis. Cross-species: human sepsis concordance not established. Clinical: preclinical context.	(58)

## 8 DC–T-cell instruction and bidirectional feedback

The functional importance of an altered DC compartment depends on whether antigen presentation, costimulation, cytokine output, checkpoint engagement, migration, and synapse formation change T-cell responses. DC depletion and residual dysfunction may converge on similar adaptive outcomes, whereas regulatory-like programming can redirect rather than simply weaken instruction. The evidence is strongest when a defined DC perturbation changes an antigen-specific or *in vivo* T-cell outcome, and weaker when a blood phenotype or transcriptomic program is used to infer that outcome.

Human sepsis studies establish altered inputs to T-cell priming more securely than they establish direct synapse failure. Blood DC-enriched populations show altered phenotype, subset distribution, and impaired ex vivo response relevant to antigen presentation and costimulation (10, 11). APC PD-1/PD-L1 expression provides an outcome-associated checkpoint context but does not isolate a DC-specific effect (45). A non-sepsis human blood DC study and an experimental receptor–ligand perturbation study help define how DC phenotype can influence T-cell activation, but they cannot replace sepsis-specific antigen-presentation assays (24, 25). The human evidence therefore supports disrupted APC/DC inputs and risk context, not the claim that DCs alone cause the broad T-cell dysfunction observed in sepsis.

Mouse perturbational studies demonstrate clearer DC-to-T-cell directionality. Polymicrobial sepsis reduced DC number and function and directly impaired primary CD8 T-cell responses *in vivo* (37). Reconstitution or adoptive transfer of DCs reversed components of long-term immunosuppression, reduced inhibitory or regulatory T-cell differentiation, and improved survival in murine models (26, 59). These experiments establish that manipulation of the DC compartment can change adaptive immune output in mice. They do not resolve all contributions of DC abundance versus residual-cell quality, and they do not establish feasibility or efficacy of DC transfer in human sepsis.

DC-derived cytokines and regulatory-like programs define complementary modes of T-cell instruction. Epigenetic suppression of IL-12 after severe innate activation reduced Th1-promoting capacity, providing a functional mechanism for weakened type 1 instruction (27). In contrast, the mouse mregDC-like and MMP8-associated tolerogenic programs showed that altered DC states may actively bias CD4 T-cell or regulatory outputs rather than remain inert (15, 28). The latter evidence remains preclinical and does not prove a conserved human mregDC-to-Treg axis. Cytokine loss, checkpoint context, and regulatory-like activity should therefore be measured separately instead of merged under a generic suppressive label.

The pDC–antiviral circuit is compartment- and time-specific. Human studies report changing circulating pDC abundance and survival-associated pDC percentages, but do not directly test lung recruitment or antiviral priming (29, 30). In a mouse post-sepsis viral-pneumonia model, IL-3 increased lung pDC recruitment and improved T-cell priming, providing perturbational support for a late antiviral vulnerability/rescue circuit (31). This result is not interchangeable with acute IL-3-driven inflammatory amplification and should not be extrapolated to an IL-3 indication in patients. It instead identifies a testable relation among pDC state, tissue localization, antiviral T-cell function, and secondary infection.

T-cell-to-DC marrow feedback is directionally distinct from DC-to-T-cell instruction. TLR2-induced CD8 T-cell deactivation altered DC differentiation in bone marrow during sepsis, indicating that adaptive dysfunction can modify the future DC supply (36). By contrast, impaired primary CD8 responses after septic DC damage concern the effect of DCs on T cells in peripheral or lymphoid immune responses (37). The two findings are compatible because they operate in opposite directions and different compartments; neither should be used to imply a closed, self-sustaining loop in patients without longitudinal and perturbational evidence.

**Figure 3:**
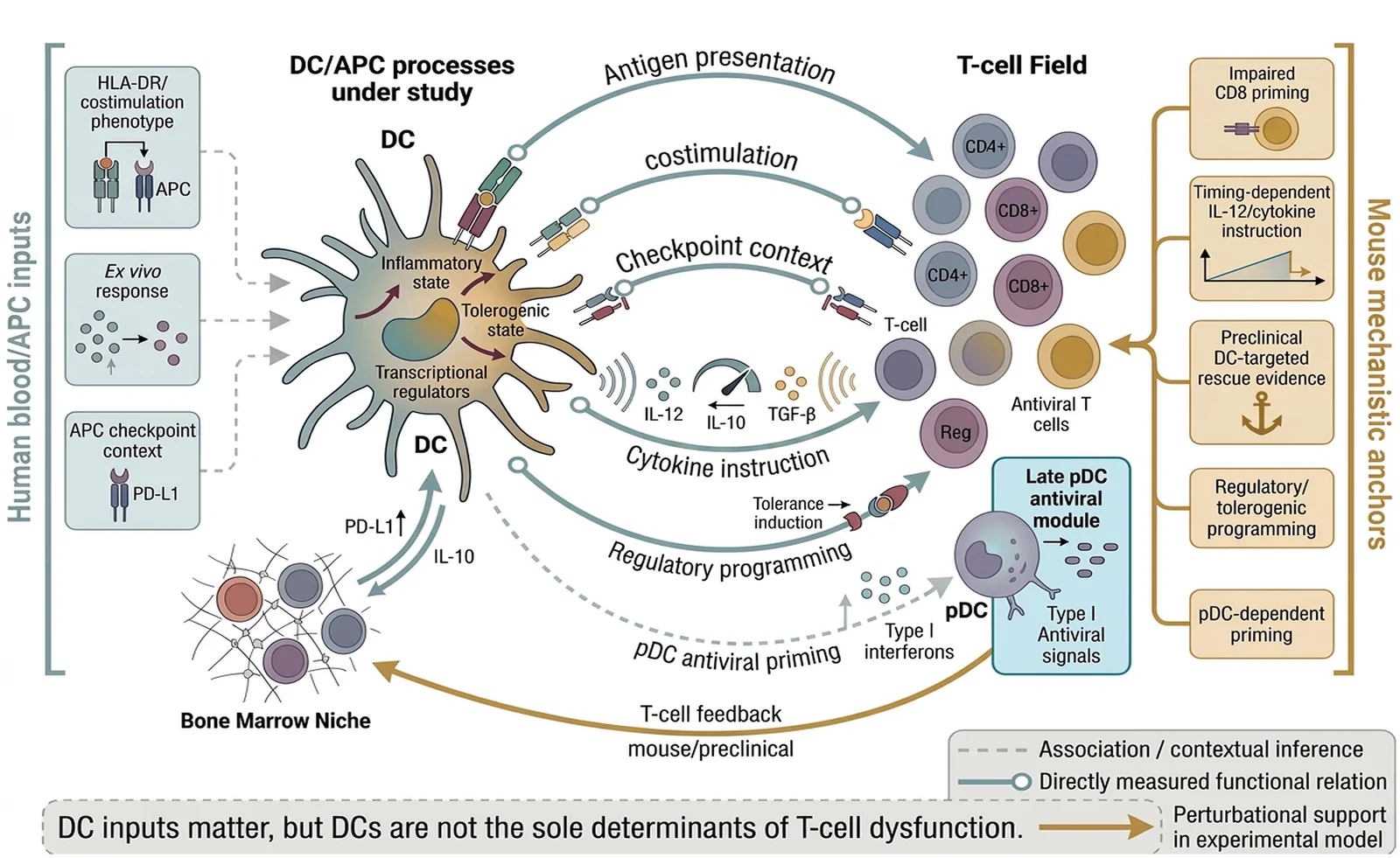
Evidence-bounded DC–T-cell circuitry and immune instruction. Human blood/APC inputs at left include HLA-DR/costimulation phenotype, ex vivo response, and APC checkpoint context. Central DC/APC processes under study comprise antigen presentation, costimulation, checkpoint context, cytokine instruction, regulatory programming, pDC antiviral priming, and T-cell feedback to the bone-marrow niche. The T-cell field and mouse mechanistic anchors distinguish altered CD4+, CD8+, regulatory T-cell, and antiviral outputs from preclinical evidence for impaired CD8 priming, timing-dependent IL-12 instruction, DC-targeted rescue, regulatory/tolerogenic programming, and pDC-dependent priming. Gray dashed lines denote association or contextual inference, teal lines with open circles denote directly measured functional relationships, and ochre arrows denote perturbational support in experimental models. These encodings should not be interpreted as equivalent evidence or as proof that DCs are the sole determinants of sepsis-associated T-cell dysfunction. APC, antigen-presenting cell; DC, dendritic cell; HLA-DR, human leukocyte antigen-DR; IL, interleukin; PD-L1, programmed death-ligand 1; pDC, plasmacytoid dendritic cell; TGF-β, transforming growth factor beta.

Overall, DC–T-cell communication is bidirectional and evidence-dependent. Human data support altered blood APC/DC inputs, whereas mouse *in vivo* and ex vivo studies more directly connect defined DC perturbations to CD8, Th1, regulatory, or antiviral T-cell outputs. **Figure 3**; **Table 3** are therefore interpreted by cellular identity, species, compartment, study design, functional validation, and perturbational support rather than as a single directional mechanism. Direct human tests should combine identity-resolved DC sorting, antigen-processing or presentation assays, cytokine measurements, matched T-cell co-culture, and clinically relevant outcomes.

**Table 3:** DC–T-cell communication axes, evidence direction, and functional consequences.

Circuit axis/evidence direction	Identity class	Species/compartment	Design and support profile	Functional consequence, inference, and boundary	Specific refs
Human antigen-presentation/costimulation inputs	Class 2 — DC-enriched or historically defined population	human; blood	observational flow cohorts; ex vivo response in one cohort. Replication: separate cohorts support altered inputs; longitudinal: serial sampling in some cohorts; functional: ex vivo responsiveness measured; antigen-specific synapse not measured; causal: none.	Supported: Supports altered DC-enriched abundance, HLA-DR/costimulation context, and responsiveness relevant to T-cell priming. Boundary: Direct human antigen-specific DC–T-cell synapse failure remains under-validated. Cross-species: not assessed. Clinical: human observational/ex vivo research evidence.	(10, 11)
Human APC checkpoint context	Class 2 — DC-enriched or historically defined population	human; blood	prospective APC PD-1/PD-L1 cohort. Replication: no matched independent DC-specific replication assigned; longitudinal: early-sepsis sampling; functional: phenotype/clinical association; no DC-specific T-cell assay; causal: none.	Supported: Supports checkpoint-adjacent risk context at the APC interface. Boundary: Does not prove a DC-specific checkpoint mechanism or justify checkpoint therapy. Cross-species: not DC-specific concordance. Clinical: human observational discovery.	(45)
Human RNA-defined APC/DC input state	Class 3 — transcriptomically inferred APC/DC-like state	human; blood	single-cell observational study with marker confirmation. Replication: no matched functional replication assigned; longitudinal: no repeated-participant trajectory established; functional: marker/protein confirmation; no antigen-specific function; causal: none.	Supported: Supports an antigen-presentation-low/anergic-like APC/DC input state. Boundary: RNA state is not direct evidence of impaired synapse formation or T-cell priming. Cross-species: not assessed. Clinical: human observational discovery.	(12)
Primary CD8 T-cell priming after polymicrobial sepsis	Class 2 — DC-enriched or historically defined population	mouse; systemic/spleen	*in vivo* antigen-targeting and Flt3L intervention. Replication: no matched independent replication assigned; longitudinal: post-sepsis window; functional: direct antigen-specific CD8 response; causal: DC-targeted intervention.	Supported: Supports DC-to-T-cell causal directionality for primary CD8 priming in mice. Boundary: Mechanistic anchor, not human proof or treatment guidance. Cross-species: no direct human synapse confirmation. Clinical: preclinical discovery.	(37)
Post-sepsis DC reconstitution/transfer and adaptive recovery	Class 2 — DC-enriched or historically defined population	mouse; lung/systemic	DC transfer/reconstitution experiments in secondary-challenge models. Replication: distinct proof-of-principle studies; not matched replication; longitudinal: post-sepsis challenge windows; functional: T-cell differentiation, host defense, and survival outcomes; causal: adoptive DC transfer.	Supported: Supports that manipulating the DC compartment can alter adaptive outputs in mice. Boundary: Does not resolve abundance versus residual-cell quality or support human DC transfer. Cross-species: no human feasibility/efficacy evidence. Clinical: preclinical proof-of-principle.	(59, 26)
DC-derived IL-12 and type-1 instruction	Class 2 — DC-enriched or historically defined population	mouse; DC culture/systemic	mechanistic cytokine-regulation studies. Replication: related studies address distinct upstream contexts; longitudinal: context/time dependent; functional: IL-12 and T-cell-instruction assays; causal: pathway/endocrine perturbation.	Supported: Supports DC cytokine output as a determinant of Th1/CD8 instruction within models. Boundary: More cytokine is not automatically beneficial; human treatment relevance is unproven. Cross-species: human conservation unproven. Clinical: preclinical discovery.	(27, 53)
Mouse mregDC-like T-cell instruction	Class 4 — preclinical regulatory/mregDC-like program without established human homolog	mouse; spleen/multi-compartment	serial cross-sectional multimodal study with sorted-cell co-culture and inhibitors. Replication: no matched independent replication; longitudinal: 8, 24, 72 h in different animals; functional: direct sorted-cell CD4 co-culture; mixed functional pattern; causal: pathway inhibitors; no selective *in vivo* depletion.	Supported: Supports exploratory regulatory-like T-cell instruction *in vitro*. Boundary: Not a conserved human mregDC–Treg axis, same-cell transition, or universal endpoint. Cross-species: no validated human sepsis homolog. Clinical: preclinical discovery.	(15)
MMP8-associated tolerogenic instruction	Class 2 — DC-enriched or historically defined population	mouse; spleen/BMDC; late sepsis	knockout/knockdown, adoptive transfer, and pathway experiments. Replication: no matched independent replication assigned; longitudinal: late model window; functional: Treg/Th1 outputs and survival/organ outcomes; causal: MMP8 perturbation; NF-κB p65/β-catenin-associated support.	Supported: Supports a distinct MMP8-linked tolerogenic DC program affecting T-cell outputs in mice. Boundary: Do not equate with mregDC-like identity or infer a human target. Cross-species: human DC concordance absent. Clinical: preclinical discovery.	(28)
Post-sepsis lung pDC antiviral priming	Class 2 — DC-enriched or historically defined population	mouse; lung	CLP plus secondary viral-pneumonia perturbation. Replication: no matched independent replication assigned; longitudinal: defined late challenge window; functional: lung pDC recruitment and antiviral T-cell priming; causal: IL-3/genetic-intervention support.	Supported: Supports a compartment-specific pDC-to-T-cell antiviral circuit in mice. Boundary: Not a clinical IL-3 recommendation and not interchangeable with acute IL-3 inflammation. Cross-species: human blood pDC associations are not functionally matched. Clinical: preclinical discovery.	(31)
T-cell-to-DC marrow feedback	Class 2 — DC-enriched or historically defined population	mouse; bone marrow	polymicrobial-sepsis mechanistic study. Replication: no matched independent replication assigned; longitudinal: defined post-sepsis windows; functional: DC differentiation and T-cell-state assays; causal: TLR2/CD8 perturbational support.	Supported: Supports reverse directionality: CD8 T-cell state can shape future DC differentiation. Boundary: Distinct from DC-to-T-cell priming; not a human marrow claim. Cross-species: no human marrow concordance. Clinical: preclinical discovery.	(36)
Non-sepsis human DC/receptor–ligand circuitry context	Class 2 — DC-enriched or historically defined population	human; blood/*in vitro*; not sepsis-specific	human DC phenotype and receptor–ligand studies. Replication: independent contextual studies; longitudinal: not sepsis longitudinal evidence; functional: T-cell activation/receptor–ligand assays; causal: study-specific experimental manipulation.	Supported: Defines plausible DC–T-cell interaction variables for future sepsis assays. Boundary: Cannot substitute for sepsis-specific antigen-presentation or synapse experiments. Cross-species: not a sepsis concordance test. Clinical: context only.	(24, 25)

## 9 DC-informed monitoring within multimodal immune stratification

DC-informed monitoring could add cell-specific information to broader immune assessment, but it is not a validated DC-only endotype or treatment-selection system. Each candidate readout should be described with the approved ten-domain profile: cellular identity confidence, species, compartment, design, replication, longitudinal support, functional validation, perturbational support, cross-species concordance, and clinical relevance/readiness. This approach prevents an accessible blood measurement from being mistaken for a tissue mechanism or a discovery signature from being presented as a bedside assay.

The most directly measurable DC-related signals are circulating abundance, broad subset distribution, phenotype, and ex vivo responsiveness. Lower blood DC counts were associated with fatal outcome, persistent reduction was associated with ICU-acquired infection, and flow studies documented abnormalities in circulating DC-enriched populations (8, 9, 11). Phenotype and stimulation assays showed dysfunction beyond numerical depletion (10). These human observational and ex vivo findings justify prospective research monitoring of count and residual function together, but they have not established a reproducible DC-only threshold, independent clinical utility, or a treatment trigger.

DC measurements must be benchmarked against established broader immune-monitoring approaches. Monocyte HLA-DR has a larger sepsis literature as a marker of immunoparalysis and secondary-infection risk, but it is a monocyte measure rather than a DC biomarker (60–62). An HLA-based transcriptomic classifier supplies one broader molecular comparator (63). Disease-stage and gene-expression subgroup studies provide additional whole-blood stratification comparators (5–7). High-dimensional mass cytometry contributes multicellular protein/phenotype context (23). DC-informed readouts should be tested for incremental value beyond these approaches rather than relabelling them as DC-specific.

Transcriptomic APC/DC-like states can enrich monitoring hypotheses but currently remain discovery-level or research-assay candidates. Broad human blood single-cell profiling supplies immune-architecture context rather than a DC-specific assay (4). More focused analyses nominate anergic-like APC/DC programs and prognosis-associated immune-state differences (12, 13). Pediatric and persistent-sepsis datasets show that signatures also vary by population and window (39, 40). Translation requires stable marker sets, standardized sampling and processing, replication in independent cohorts, conversion to feasible assays, and linkage to protein phenotype, ex vivo function, and outcomes. An RNA-defined state should not be used as a functional endotype until those steps are completed.

Developmental supply and tissue localization are biologically important but difficult to monitor from blood. Mouse marrow studies support altered DC generation, emergency monopoiesis, and T-cell feedback onto differentiation (14, 34, 36). A pediatric human blood/progenitor study offers indirect supply-side measurements and *in vitro* differentiation, not direct patient-marrow monitoring (16). Murine splenic depletion and post-sepsis lung pDC recruitment demonstrate tissue events that are not directly visible in circulation (31, 32). Candidate human surrogates—such as serial subset recovery, progenitor-associated modules, or paired monocyte/DC output patterns—remain hypotheses until they are compared with direct or tissue-adjacent measurements. They must not be called marrow-output or redistribution biomarkers without validation.

pDC monitoring provides a focused example of multimodal design. Human blood studies support serial assessment of pDC count or state, while the mouse lung study identifies antiviral T-cell priming and secondary viral pneumonia as plausible functional outcomes (29–31). A rigorous study would pair blood pDC identity and abundance with interferon responsiveness, virological outcomes, sampling time, and—when clinically justified—respiratory-compartment data. Until human compartmental and functional concordance is shown, this remains a research question rather than an antiviral-risk classifier.

**Figure 4:**
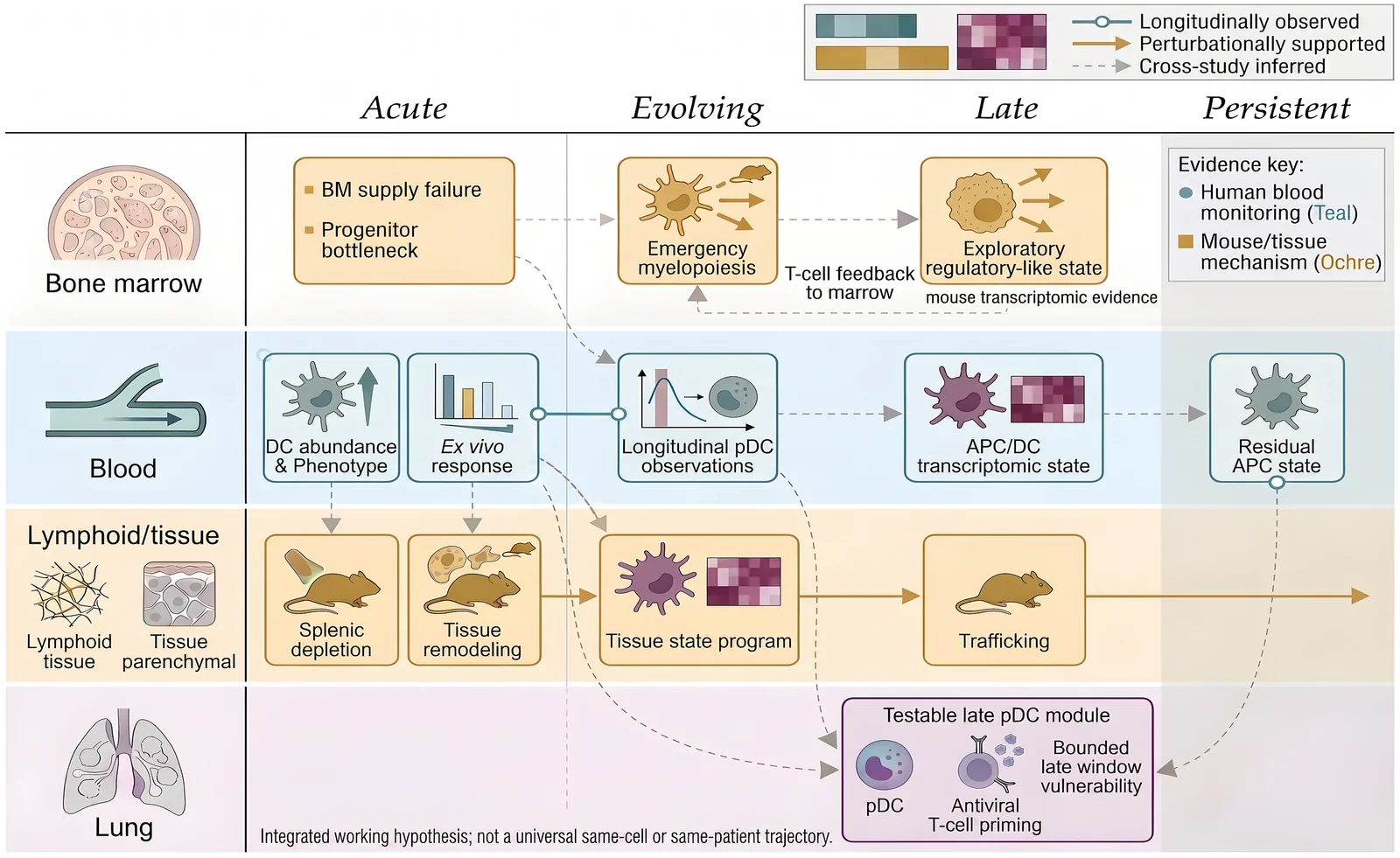
DC-associated candidate readouts, validation domains, and translational boundaries. Existing monitoring approaches at left—monocyte HLA-DR, broad transcriptomic subgroups, HLA/classifier-style layers, and immunoparalysis stratification—are comparators and are not relabeled as DC-specific. Candidate DC-associated research readouts include abundance/depletion or flux, cDC1/cDC2/pDC phenotype, ex vivo response, checkpoint context, APC/DC transcriptomic states, pDC antiviral/IFN state, and supply inference. The right panel lists research hypotheses requiring validation: bone-marrow supply failure, a late pDC antiviral-risk window, regulatory/tolerogenic programming, DC rescue concepts, and organelle-stress/metabolic mechanisms. Gray dotted links indicate contextual or testable relationships, not causal transitions. The lower boxes are separate validation domains—research-grade measurement, functional/orthogonal validation, causal-preclinical evidence, and prospective clinical validation—not an ordinal readiness score. No DC-only endotype, treatment trigger, validated DC-directed therapy, or treatment recommendation is established. APC, antigen-presenting cell; BM, bone marrow; cDC, conventional dendritic cell; HLA-DR, human leukocyte antigen-DR; IFN, interferon; pDC, plasmacytoid dendritic cell.

A practical DC-informed monitoring study should combine serial abundance, identity-resolved phenotype, ex vivo function, APC checkpoint context, and selected transcriptomic modules, then test whether those measurements add information beyond monocyte HLA-DR, broader immune subgrouping, and routine clinical variables. The protocol should prespecify sampling windows, compartments, technical reproducibility, outcomes, and the ten evidence domains used for interpretation. **Figure 4**; **Table 4** organize candidate readouts and their boundaries; they do not confer validation. No DC-only endotype, bedside biomarker, or intervention trigger is currently established.

**Table 4:** DC-informed research monitoring, comparator readouts, and evidence boundaries.

Readout role	Identity class	Species/compartment	Design and validation profile	Potential use and explicit boundary	Specific refs
DC candidate — circulating DC-enriched abundance	Class 2 — DC-enriched or historically defined population	human; blood	observational flow cohorts. Replication: separate cohorts support association; methods/populations vary; longitudinal: serial support in some cohorts; functional: none or phenotype only; causal: none.	Supported: May support longitudinal abundance/recovery tracking when assay definitions and timing are explicit. Boundary: Not a DC-only endotype, validated bedside biomarker, or treatment trigger. Cross-species: not assessed. Clinical: human observational discovery/research-monitoring candidate.	(8, 9, 11)
DC candidate — phenotype plus ex vivo responsiveness	Class 2 — DC-enriched or historically defined population	human; blood	prospective flow cohort with stimulation assays. Replication: no matched independent functional replication established; longitudinal: baseline/day-28 sampling; functional: direct ex vivo cytokine-response assays; causal: none.	Supported: May distinguish residual-cell deactivation from count loss in research cohorts. Boundary: Not tissue proof, antigen-specific synapse proof, or a treatment trigger. Cross-species: not assessed. Clinical: human ex vivo research evidence.	(10)
DC/APC candidate — checkpoint-adjacent phenotype	Class 2 — DC-enriched or historically defined population	human; blood	prospective APC PD-1/PD-L1 cohort. Replication: no matched independent DC-specific replication assigned; longitudinal: early sampling; functional: phenotype/clinical association; no DC-specific function; causal: none.	Supported: May provide APC checkpoint context within multimodal immune stratification. Boundary: Not a DC-specific checkpoint biomarker or therapy rationale. Cross-species: not DC-specific. Clinical: human observational discovery.	(45)
DC candidate — anergic-like APC/DC transcriptomic state	Class 3 — transcriptomically inferred APC/DC-like state	human; blood	single-cell observational study with marker confirmation. Replication: no matched independent functional replication assigned; longitudinal: no repeated-participant trajectory established; functional: marker/protein confirmation; no direct antigen-specific function; causal: none.	Supported: May support assay-development work for an antigen-presentation-low APC/DC state. Boundary: RNA state alone is not direct function, a clinical endotype, or a treatment trigger. Cross-species: not assessed. Clinical: human observational discovery.	(12)
DC-associated discovery — broader single-cell immune states	Class 3 — transcriptomically inferred APC/DC-like state	human; blood	single-cell observational cohorts/reanalyses. Replication: contextual convergence across distinct cohorts; not matched state replication; longitudinal: study-dependent; functional: omics; DC-resolved function generally absent; causal: none.	Supported: May identify DC-associated modules for prospective validation. Boundary: Broad immune states must not be presented as DC-specific biomarkers. Cross-species: contextual only. Clinical: human observational discovery.	(4, 13)
DC candidate — progenitor/supply measurements	Class 1 — phenotypically/ontogenetically well-defined DC	human; blood and HSPC culture	pediatric cohort plus *in vitro* differentiation. Replication: no matched external replication assigned; longitudinal: days 1–7 sampling; not marrow trajectory; functional: flow, apoptosis, differentiation, and cytokine assays; causal: *in vitro* mediator perturbation.	Supported: May motivate paired mature-DC/progenitor monitoring in defined research cohorts. Boundary: Not a validated surrogate of marrow output or a treatment trigger. Cross-species: human evidence; marrow compartment remains indirect. Clinical: human mechanistic/ex vivo research evidence.	(16)
DC research hypothesis — mouse marrow supply modules	Class 2 — DC-enriched or historically defined population	mouse; bone marrow	mechanistic/perturbational marrow studies. Replication: related studies; no matched human replication; longitudinal: defined experimental windows; functional: differentiation/output assays; causal: model-specific perturbation.	Supported: Provides mechanisms to nominate, not validate, human supply-side measurements. Boundary: Not direct evidence for a clinical human marrow assay. Cross-species: human concordance not established. Clinical: preclinical discovery.	(14, 34, 36)
DC research hypothesis — pDC antiviral-risk module	Class 2 — DC-enriched or historically defined population	mouse with separate human context; lung; human context is blood	mouse secondary-viral challenge plus separate human observational pDC studies. Replication: cross-context support; not matched replication; longitudinal: defined mouse late window; human sampling study-dependent; functional: mouse pDC recruitment/T-cell priming; human rows lack matched function; causal: IL-3/genetic support in mouse model.	Supported: Supports prospective study of pDC count/state alongside viral outcomes in a prespecified late window. Boundary: Not a validated antiviral-risk biomarker or clinical IL-3 recommendation. Cross-species: species/compartment concordance incomplete. Clinical: preclinical discovery with human observational context.	(31, 29, 30)
Comparator — monocyte HLA-DR	N/A — comparator is not DC-specific	human; blood	established broader immune-monitoring research literature. Replication: multiple independent cohorts/assay studies; longitudinal: serial use reported in the literature; functional: flow phenotype; not DC function; causal: interventional utility remains context-dependent.	Supported: Benchmarks whether DC measures add information beyond an established broader cellular readout. Boundary: Must not be relabelled as DC-specific; it is not by itself a universal treatment trigger. Cross-species: not DC-specific. Clinical: broader human immune-monitoring comparator.	(60–62)
Comparator — whole-blood immune subgroups/classifiers	N/A — comparator is not DC-specific	human; blood	bulk transcriptomic classification/validation studies. Replication: independent cohorts and external validation vary by classifier; longitudinal: sampling schedules vary; functional: classifier validation; not DC-resolved function; causal: none for DC causality.	Supported: Benchmarks incremental value of DC-resolved measurements against broader immune subgrouping. Boundary: Do not call a whole-blood classifier a DC biomarker without DC-resolved validation. Cross-species: not DC-specific. Clinical: broader human stratification comparator.	(5–7)
Comparator — broader gene-expression/immune signatures	N/A — comparator is not DC-specific	human; blood	gene-expression signature and immune-stratification studies. Replication: study-specific external validation; longitudinal: sampling schedules vary; functional: molecular classifier performance; not DC-specific function; causal: none for DC causality.	Supported: Provides a second benchmark for added predictive or mechanistic value. Boundary: Cannot be rebranded as DC-specific and does not validate a DC-only endotype. Cross-species: not DC-specific. Clinical: broader human stratification comparator.	(63, 64)

## 10 Therapeutic hypotheses and evidence boundaries

Current therapeutic implications are research hypotheses derived from specific evidence profiles, not recommendations. Any proposed intervention must identify the upstream context, the measurable DC process, the intended functional consequence, the species and compartment supporting the claim, the timing window, and the safety boundary. A signal confined to transcriptomic discovery, a pathway effect demonstrated only *in vitro*, or a survival benefit in a mouse model cannot by itself define a human treatment population.

The IL-3/pDC axis demonstrates the need for timing- and state-aware design. IL-3 enhanced lung pDC recruitment and antiviral T-cell priming in post-sepsis viral pneumonia, supporting a preclinical late-rescue hypothesis (31). In a different acute context, IL-3 amplified inflammatory myelopoiesis and inflammation (46). A responsible translational program would first establish whether a patient has pDC-related antiviral dysfunction, define the secondary-infection window, monitor inflammatory risk, and specify stopping rules. The evidence does not support general IL-3 administration or a DC-based treatment trigger.

DC reconstitution and adoptive transfer provide population-level causal proof in mice. Restoration of the DC compartment reversed components of long-term post-sepsis immunosuppression, and bone-marrow-derived DC transfer changed inhibitory or regulatory T-cell differentiation and improved survival in murine polymicrobial sepsis (26, 59). These results justify studying which DC functions are rescue-limiting. They do not establish manufacturing feasibility, cell identity equivalence, biodistribution, dose, safety, timing, or target-patient selection for human sepsis, and they should not be presented as near-term clinical strategies.

Regulatory-like and checkpoint findings suggest stratification questions, not ready targets. MMP8 perturbation altered late tolerogenic DC programming in mouse polymicrobial sepsis, and the mregDC-like atlas identified a preclinical regulatory program with bounded functional support (15, 28). In patients, APC PD-1/PD-L1 expression is associated with severity and outcome context, but does not establish DC-specific checkpoint dependence or predict benefit from checkpoint intervention (45). Future studies should determine identity, timing, reversibility, and off-target inflammatory risk before considering pathway-directed intervention.

Organelle, autophagy, and metabolic pathways remain translationally distant. mtDNA–STING and miR-146a-5p/ATG7 studies nominate distinct DC immunoparalysis and glycolytic-remodeling mechanisms (48, 49). PINK1 and Sestrin2 studies address mitochondrial quality control, endoplasmic-reticulum-stress apoptosis, or pyroptosis rather than one shared pathway (41, 42, 50). RETREG1/FAM134B-mediated ERGICphagy and NUFIP1-mediated ribophagy are separate, recent preclinical mechanisms (51, 52). Their value is to nominate measurable mechanisms and discriminating experiments. Advancement would require independent replication, validation in identity-resolved human DCs, evidence that each pathway is not merely a general injury response, a clinically feasible readout, and a safety case for intervention.

Across these hypotheses, progression toward clinical testing should be governed by the approved appraisal domains rather than a separate readiness ladder. At minimum, a candidate requires a defined DC population, a reproducible human assay, longitudinal association in the intended compartment and window, direct functional validation, relevant perturbational support, comparison with broader immune monitoring, and prespecified safety limits. The current literature does not satisfy this combination for any DC-directed intervention. No DC-only endotype, treatment trigger, or DC-directed therapy is validated.

## 11 Conflicting evidence, limitations, and validation priorities

The principal limitation is that different studies answer different biological questions. Human cohorts mainly describe blood abundance, phenotype, ex vivo response, checkpoint context, or transcriptomic state. Mouse and *in vitro* studies provide access to marrow, spleen, lung, tissue architecture, and perturbation. These evidence types are complementary, but apparent disagreement should be interpreted through the relevant identity, species, compartment, time, design, and functional assay rather than resolved by choosing one model or constructing a universal sequence.

Human blood and murine marrow, spleen, or lung evidence address different biological levels. A low circulating DC count is established in human septic shock cohorts (8), whereas splenic apoptosis and depletion were measured in mice (32). Altered marrow generation and post-sepsis lung pDC recruitment represent two additional, experimentally distinct compartments (14, 31). Review-level synthesis supports considering these processes together but cannot establish that any one caused the human blood result (65). Likewise, blood and tissue measurements need not move in parallel because trafficking, retention, local survival, and sampling differ. Cross-species validation should compare matched identities and functions, while human studies should add feasible tissue-adjacent sampling rather than treat blood as a proxy.

Early inflammatory effects and late or persistent immune suppression are not mutually exclusive, but neither forms a mandatory chronological sequence. IL-3 amplified acute inflammation in an experimental sepsis setting (46). Separate studies addressed sustained impairment of marrow DC supply, late tolerogenic programming, and persistent human immune-state abnormalities (14, 28, 39). The IL-3 literature further exemplifies context dependence because acute inflammatory amplification and late pDC-associated antiviral rescue arose in different models and windows (31, 46). Future studies must prespecify onset-relative time, recovery state, ongoing infection, organ injury, and treatment exposure.

DC depletion, dysfunction of residual cells, redistribution, and impaired production are competing or coexisting explanations for an abnormal compartment, not interchangeable labels. Human blood-count studies measure abundance in one compartment (8), whereas ex vivo stimulation assays measure retained responsiveness of residual blood DC-enriched populations (10). Murine tissue histology and apoptosis assays address local splenic loss (32), and marrow differentiation experiments address supply (14). No cited study directly quantifies paired-compartment redistribution in patients. Validation should therefore combine serial counts with identity-resolved phenotype, direct function, supply indicators, and tissue-adjacent measurements rather than infer cause from one readout.

Transcriptomic state and functional phenotype must also remain distinct. Human anergic-like APC/DC programs and the mouse mregDC-like program identify candidate biology, but RNA expression does not itself measure antigen processing, peptide presentation, cytokine secretion, migration, or T-cell instruction (12, 15). The Yao et al. study contains important within-study protein, spatial, co-culture, and inhibitor support, yet remains serial cross-sectional and lacks a validated human sepsis homolog. Future omics studies should incorporate matched protein phenotyping, sorted-cell functional assays, perturbation where appropriate, repeated-participant sampling, and clinically relevant outcomes.

Patient and model heterogeneity further limits generalization. Adult and pediatric disease, immune competence, infection source and pathogen, surgical versus medical critical illness, acute shock versus chronic critical illness, secondary infection, organ failure, and treatment exposure can produce different DC measurements. Pediatric transcriptomic studies define age-specific contexts rather than adult-equivalent endpoints (40, 66). Late-sepsis, recovery, and persistent-immune-dysfunction studies likewise address different populations and cellular scopes (38, 39, 67). Intervention studies must enrich on a measurable state and window, retain broader inflammatory and immunosuppressive comparators, and include stopping rules because immune stimulation or suppression may be harmful outside the intended context.

The validation agenda follows directly from these conflicts. Prospective studies should use standardized panels that distinguish phenotypically defined DC subsets from historical DC-enriched populations and transcriptomically inferred APC/DC-like states. Repeated blood samples should be collected across prespecified windows, and abundance should be paired with protein phenotype, antigen-presentation or cytokine assays, and clinical outcomes. Respiratory, infection-source, marrow-adjacent, or other tissue information should be added when feasible. Experimental models should test directionality for both DC-to-T-cell instruction and T-cell-to-DC marrow feedback and prioritize mechanisms with independent replication and human concordance. Candidate monitoring strategies must demonstrate incremental value beyond monocyte HLA-DR, transcriptomic immune subgrouping, and routine clinical variables before interventional use is considered.

## 12 Conclusion

Sepsis-associated DC dysfunction comprises separable but potentially coexisting abnormalities in abundance, developmental supply, survival, localization, antigen presentation, cytokine instruction, checkpoint context, regulatory-like programming, organelle stress, pDC antiviral function, and DC–T-cell communication. Human blood studies document clinically relevant depletion, phenotypic, ex vivo, checkpoint, and transcriptomic signals, whereas mouse marrow, spleen, lung, and perturbational studies provide stronger mechanistic directionality within their models. These bodies of evidence should be connected without equating blood with tissue, transcriptomic state with function, or cross-study timing with a continuous patient trajectory. DC-informed measurements may add value to longitudinal multimodal immune stratification, but they must be benchmarked against broader approaches and interpreted using the defined identity and evidence domains. No DC-only endotype, validated bedside biomarker, treatment trigger, or DC-directed therapy is established. The priority is prospective, identity-resolved, compartment-aware work that links serial abundance and molecular state to direct function, bidirectional DC–T-cell effects, clinically relevant outcomes, and explicit safety boundaries.
